# Irregular shape as an independent predictor of prognosis in patients with primary intracerebral hemorrhage

**DOI:** 10.1038/s41598-022-12536-3

**Published:** 2022-05-20

**Authors:** Chunyang Liu, Haopeng Zhang, Lixiang Wang, Qiuyi Jiang, Enzhou Lu, Chao Yuan, Yanchao Liang, Zhenying Sun, Huan Xiang, Xun Xu, Jingxian Sun, Bo Fu, Boxian Zhao, Daming Zhang, Xin Chen, Ning Wang, Lu Wang, Guang Yang

**Affiliations:** 1 Department of Neurosurgery, The First Affiliated Hospital of Harbin Medical University, Youzheng Street 23, Nangang District, Harbin, 150001 Heilongjiang Province China; 2 Institute of Brain Science, Harbin Medical University, Harbin, China; 3 Department of Urology, The Fourth Hospital of Harbin Medical University, Yiyuan Street 37, Nangang District, Harbin, 150001 Heilongjiang Province China

**Keywords:** Diseases of the nervous system, Neurological disorders, Clinical trial design

## Abstract

The utility of noncontrast computed tomography markers in the prognosis of spontaneous intracerebral hemorrhage has been studied. This study aimed to investigate the predictive value of the computed tomography (CT) irregularity shape for poor functional outcomes in patients with spontaneous intracerebral hemorrhage. We retrospectively reviewed all 782 patients with intracranial hemorrhage in our stroke emergency center from January 2018 to September 2019. Laboratory examination and CT examination were performed within 24 h of admission. After three months, the patient's functional outcome was assessed using the modified Rankin Scale. Multinomial logistic regression analyses were applied to identify independent predictors of functional outcome in patients with intracerebral hemorrhage. Out of the 627 patients included in this study, those with irregular shapes on CT imaging had a higher proportion of poor outcomes and mortality 90 days after discharge (*P* < 0.001). Irregular shapes were found to be significant independent predictors of poor outcome and mortality on multiple logistic regression analysis. In addition, the increase in plasma D-dimer was associated with the occurrence of irregular shapes (*P* = 0.0387). Patients with irregular shapes showed worse functional outcomes after intracerebral hemorrhage. The elevated expression level of plasma D-dimers may be directly related to the formation of irregular shapes.

## Introduction

Spontaneous intracranial hemorrhage (ICH), a subtype of stroke with a severe high mortality and disability rate, accounts for approximately 10–15% of all stroke patients^1–3^. Early hematoma expansion (HE), frequently accompanied by the occurrence of neurological deterioration, is associated with poor functional outcomes in patients with intracerebral hemorrhage^4^. The heterogeneity of hematoma has been verified to be associated with hematoma expansion^5,6^. Although the CT angiography (CTA) spot sign is accepted as the gold standard for judging the risk of hematoma expansion^7^, the need for a contrast agent limits its availability and poses an additional risk for some patients. Therefore, the prompt and accurate identification of patients that at high risk of developing hematoma expansion and giving them individualized treatment measures may provide benefits in functional outcomes^8,9^. Compared with CTA, noncontrast computed tomography (NCCT) is an inexpensive and widely used examination tool that is more suitable for early diagnostic applications in patients with intracerebral hemorrhage^10^. Some NCCT biomarkers of intracerebral hemorrhage have received increasing attention from researchers for their good performance in predicting the poor prognosis of patients. Morotti et al. showed good predictive performance when the irregular shape was used as an NCCT marker for judging early intracerebral hemorrhage expansion^11^. However, this study was based on unadjusted pooled estimates, there was no independent collection of real patient medical records for analysis, and the factors associated with the appearance of irregular shapes have not been explored.

Therefore, in this study, we comprehensively collected the clinical data of patients with intracerebral hemorrhage and explored whether the irregular shape could serve as an independent predictor for judging patient prognosis. In our previous studies, it was confirmed that plasma D-dimer expression levels can predict the poor prognosis of patients with intracerebral hemorrhage^12^. On this basis, we further investigated whether there was a specific correlation between the appearance of the irregular shape of the hematoma and the elevated expression level of plasma D-dimer.

## Patients and methods

### Patients

The clinical data of 782 patients with ICH admitted to the emergency neurosurgery ward of the First Affiliated Hospital of Harbin Medical University from January 2018 to September 2019 were retrospectively analyzed. The inclusion and exclusion criteria were as follows: age ≥ 18 years; CT scans performed within 24 h of admission; and standard techniques used (CA-7000 Sysmex; Dade Behring) to analyze the concentration of D-dimer. Patients without CT images or follow-up CT scans within 24 h after the initial CT examination, patients with intracranial hemorrhage due to trauma or aneurysm, and patients with infratentorial hemorrhage were excluded.

To assess the clinical status, the Glasgow Coma Scale (GCS) score was measured by a neurosurgeon at the time of patient admission. To determine the functional outcome, patients were followed up at least three months after symptom onset, and their clinical outcomes were assessed by the modified Rankin scale (mRS; poor functional outcome: mRS score 4–6; favorable functional outcome: mRS score 0–3). In addition, all patients were followed for survival status until death or the end of the study. The last date of Study period (September 30, 2020) was used as an end date. The study protocol was approved by the Institutional Review Board of The First Affiliated Hospital of Harbin Medical University. Simultaneously, all methods were carried out in accordance with the relevant regulations and guidelines of Harbin Medical University.

### Image features

The imaging data of the patients were evaluated independently by two experienced neurologists. Hematoma volumes were measured on industry-standard DICOM images using 3D Slicer 4.10.2, an open-source software (SPL, Harvard Medical School, Boston, USA). For patients with severe symptoms, continuous deterioration, and suspected intracerebral hemorrhage expansion, the noncontrast CT scans will be examined within 24 h after admission, and the presence of hematoma expansion and irregular shapes will be judged. Hematoma expansion was defined as an absolute growth > 6 mL or a relative growth > 33% in hematoma size on follow-up nonenhanced CT scans compared with the initial CT examination after admission^13^. Irregular shapes were defined as the presence of two or more connected or separated irregular hematomas at the edge of the hematoma on the axial section with the largest hematoma cross-sectional area^14^ (Supplemental Fig. 1).

### Statistical analysis

Statistical analysis and calculations were carried out using dedicated software (IBM SPSS Statistics 21.0; SPSS Inc, Armonk, NY, USA) or GraphPad Prism 8 software (version 8.0.3; GraphPad Software Inc, San Diego, CA). Baseline characteristics were compared between patients with and without hematoma expansion. Categorized variables were expressed as counts (percentages) and compared using the chi-square test or Fisher’s exact test, while continuous variables were expressed as the mean ± SD or median (interquartile range, IQR) values and compared using independent sample Student’s t test.

Variables associated with mortality at 90 days after discharge, poor prognosis, and hematoma enlargement were included in the univariate analysis. Variables with *P* values < 0.05 in the univariate analysis were included in multivariate logistic models to adjust for confounders. The scatter plot represents the relationship between irregular shape and plasma D-dimer expression levels. Statistical significance for all tests was defined as *P* < 0.05.

### Consent to participate

The need for informed consent was waived due to the retrospective nature of the study.

### Consent for publication

The need for informed consent was waived due to the retrospective nature of the study. The manuscript does not contain identifying details.

### Ethical approval

The study protocol was approved by the Institutional Review Board of The First Affiliated Hospital of Harbin Medical University. Simultaneously, all methods were carried out in accordance with the relevant regulations and guidelines of Harbin Medical University. And has therefore been performed in accordance with the ethical standards laid down in the 1964 Declaration of Helsinki and its later amendments. The need for informed consent was waived by the Institutional Review Board of The First Affiliated Hospital of Harbin Medical University due to the retrospective nature of the study.

## Results

**Figure 1:**
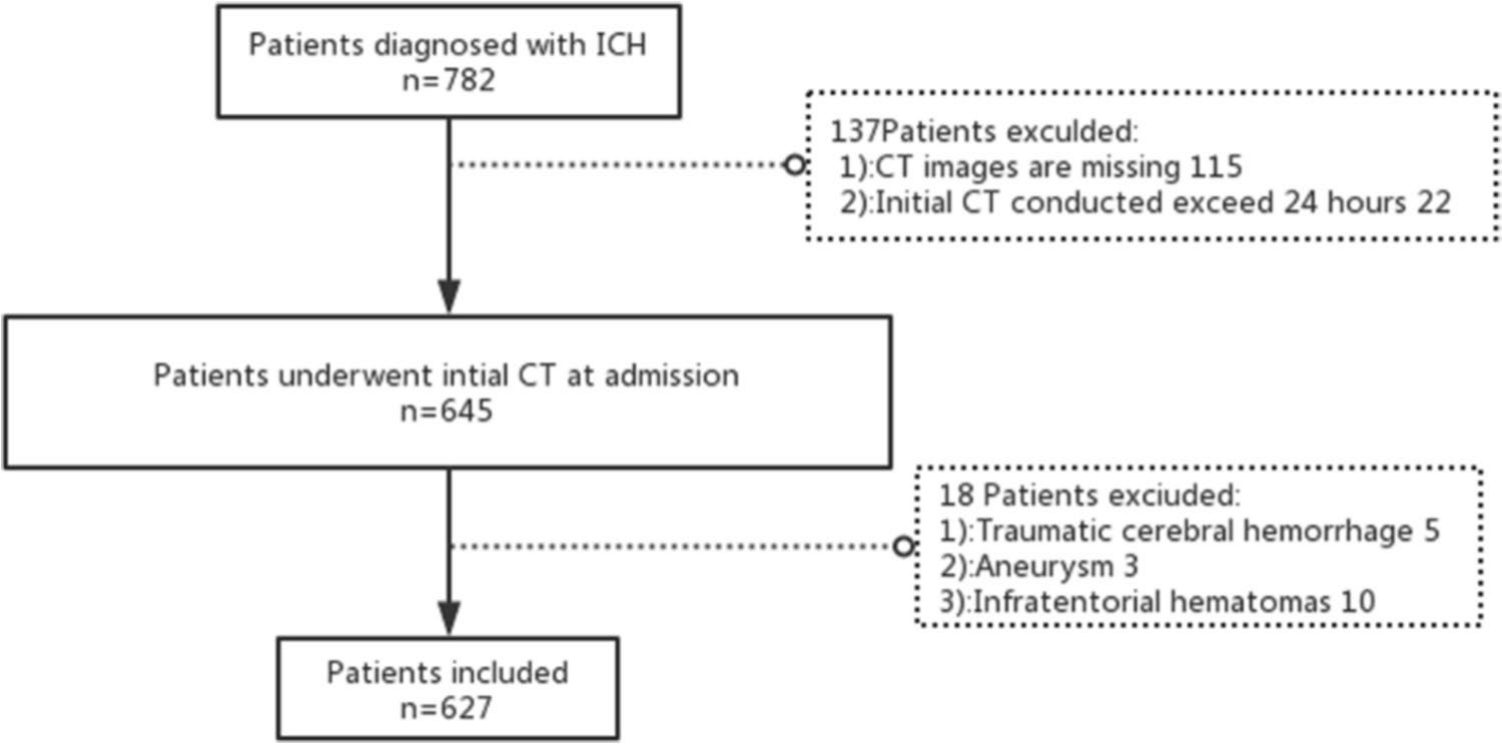
Flowchart of patient enrollment.

**Figure 2:**
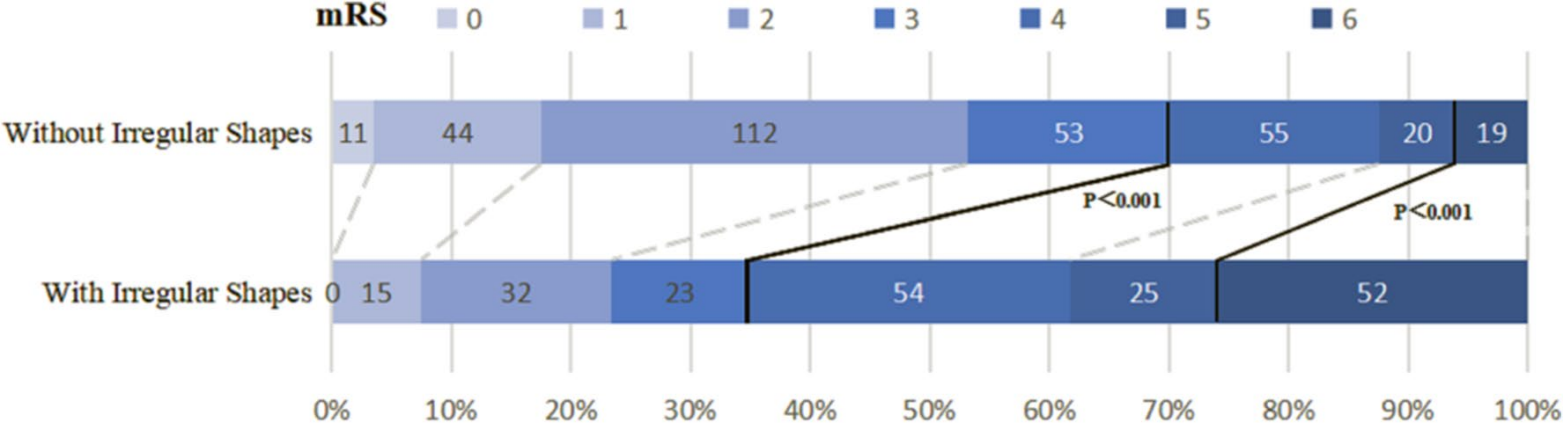
Distribution of the modified Rankin Scale (mRS) scores according to the presence or absence of irregular shapes. The bold line separates favorable (mRS, 0–3) and poor outcomes (mRS, 4–6) or survival and death.

According to the inclusion and exclusion criteria, 782 patients were included in our study. Figure 1 shows the study flow chart of patient inclusion for the analysis. There were 627 eligible patients, including 428 males and 199 females. The mean age of the patients was 57.95 years (range 18–91 years). The mean hematoma volume of all patients was 25.56 ± 27.91 mL, the mean plasma D-dimer level on admission was 1.36 ± 4.69 mg/L, and a total of 220 patients had an elevated expression level of D-dimer on admission (35.0%). Hematoma expansion was observed in 43 patients, 28 of whom presented with irregular shapes. Among the 627 patients included in this study, 248 were diagnosed with irregular shapes. The 515 patients in the study were able to continue our follow-up investigation 90 days after discharge. In patients with irregular shapes, there was a significantly higher rate of poor outcomes and mortality at 90 days after discharge (mRS = 4–6 at 3 months: positive 131/201 [65.1%] vs. negative 94/314 [29.9%], *P* < 0.001; mortality at 3 months: positive 52/201 [25.9%] vs. negative 19/314 [6.1%], *P* < 0.001; Fig. 2). Table 1 shows the comparison of clinical characteristics between patients with irregular shapes and the control group. The results showed that initial blood pressure, initial hematoma volume, hematoma expansion, presence of IVH, degree of midline shift, and elevated plasma D-dimer levels were significantly related to the occurrence of irregular shapes.

**Table 1:** Clinical characteristics related to Irregular Sign in patients with ICH.

Characteristics	Patients, no. (%)		*P* value
	Patients with irregular sign (n = 248)	Patients without irregular sign (n = 379)	
Age (years)	57.82 ± 11.57	58.04 ± 11.48	0.812
Sex (male)	165 (66.5)	263 (69.4)	0.452
History of hypertension	230 (92.7)	342 (90.5)	0.323
History of diabetes	29 (11.7)	40 (10.6)	0.664
First systolic BP (mmHg)	176.88 ± 29.73	168.33 ± 26.55	< 0.001
Heart rate	79.54 ± 19.71	80.25 ± 16.58	0.643
Temperature (℃)	36.60 ± 0.33	36.57 ± 0.24	0.198
Initial hematoma volume (ml)	27.95 (15.03–51.84)	9.98 (4.16–23.05)	< 0.001
Hematoma expansion	28 (17.5)	15 (5.3)	< 0.001
IVH	107 (43.1)	115 (30.3)	0.001
Location of ICH			0.733
Lobar	25 (10.1)	35 (9.2)	
Deep	223 (89.9)	344 ( 90.8)	
Midline shift (mm)	5.14 (4.57–5.70)	2.19 (1.87–2.50)	< 0.001
Admission INR	1.01 (0.99–1.03)	1.01 (0.99–1.03)	0.965
Admission PT (s)	11.10 (10.60–11.80)	11.20 (10.80–11.70)	0.896
Admission APTT (s)	25.60 (23.70–27.80)	25.75 (23.90–27.70)	0.683
Admission Fibrinogen (g/L)	2.85 (2.73–2.97)	2.96 (2.76–3.16)	0.417
Admission D-Dimer, > 0.55 mg/L FEU	103 (41.7)	117 (31.5)	0.009
GCS score on admission	11.01 ± 2.74	11.41 ± 2.57	0.063
Surgery	103 (41.5)	77 (20.3)	< 0.001

APTT = activated partial thromboplastin time, BP = blood pressure; GCS = Glasgow Coma Scale; ICH = intracerebral hemorrhage; INR = international normalized ratio; IVH = intraventricular hemorrhage on presentation; mRS = modified Rankin Scale; NIHSS = NIH Stroke Scale; PT = prothrombin time. Values are n (%), mean ± SD, or median (interquartile range).

**Figure 3:**
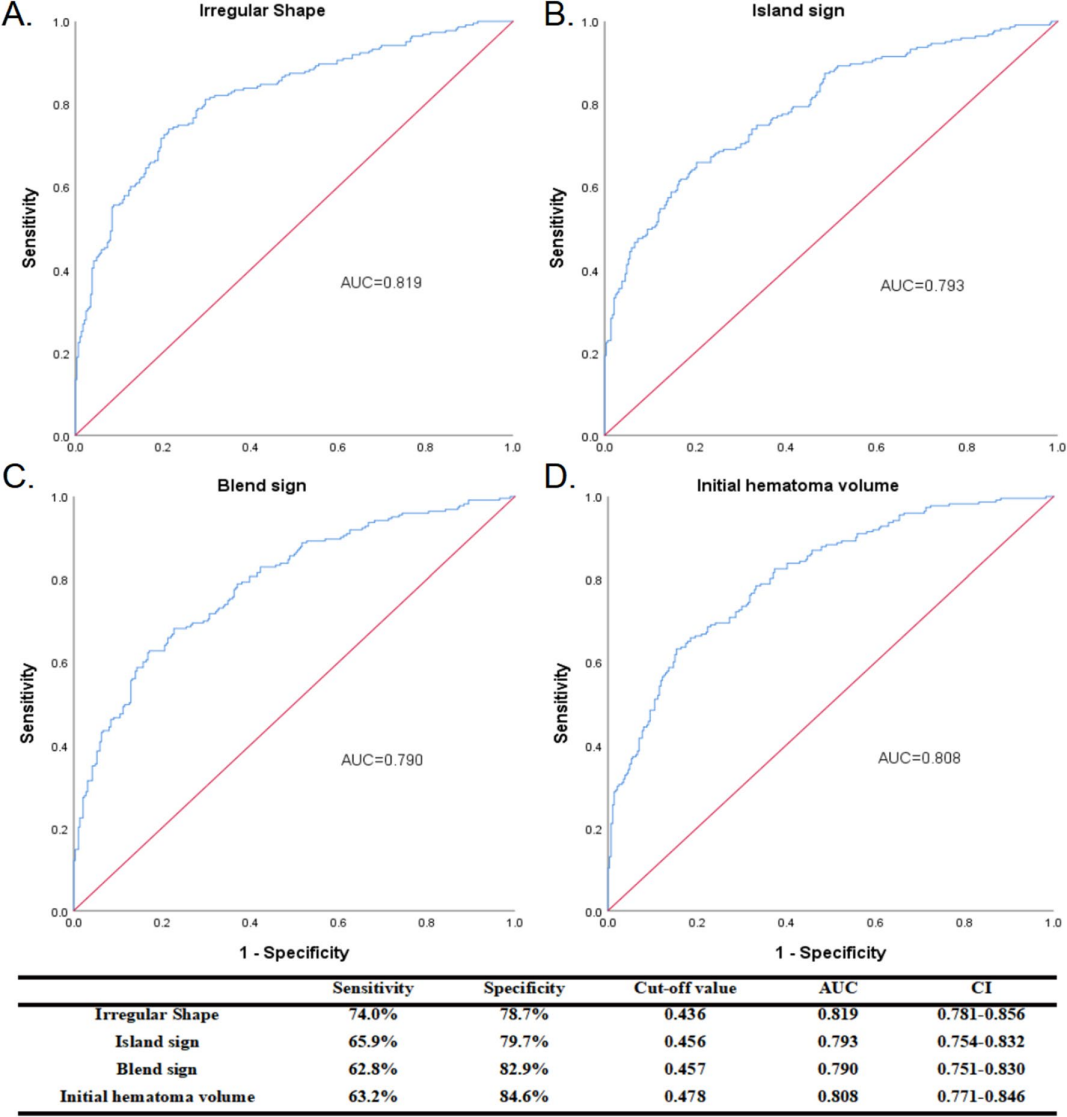
Receiver operating characteristic curves of irregular shape, island sign, blend sign, and IHV with their corresponding areas under the curve (AUCs) for predicting poor prognosis. The best cut-off points were identified with their sensitivity, specificity, and confidence interval (CI), respectively.

Univariate logistic regression analysis was performed for poor outcome and mortality after 90 days of discharge in patients with ICH, and the factors associated with the development of hematoma expansion within 24 h of admission were explored (Table 2). The analysis results indicated a significantly higher rate of early hematoma expansion in patients with irregular shapes (OR = 3.776, 95% CI 1.950–7.312, *P* < 0.001) and had a poor prognosis at 90 days (OR = 4.380, 95% CI 3.003–6.389, *P* < 0.001) and an expressively higher mortality rate (OR = 5.233, 95% CI 2.991–9.155, *P* < 0.001). The results also indicated that patients with elevated plasma D-dimer levels had a higher risk of mortality (OR = 3.518, 95% CI 2.096–5.905, *P* < 0.001) and poorer prognosis (OR = 2.809, 95% CI 1.928–4.092, *P* < 0.001). Multivariate logistic regression analyses were performed for significant variables in the univariate logistic analysis. Multivariate regression analysis showed that age (OR = 1.052, 95% CI 1.031–1.074, *P* < 0.001), first systolic blood pressure (OR = 1.008, 95% CI 1.001–1.017, *P* = 0.049), irregular shapes (OR = 3.190, 95% CI 2.007–5.070, *P* < 0.001), initial hematoma volume (OR = 1.024, 95% CI 1.009–1.039, *P* = 0.001), and intraventricular hemorrhage upon presentation (OR = 1.967, 95% CI 1.237–3.128, *P* = 0.004) independently predicted poor outcome of ICH. The irregular shape had equally good performance in independently predicting mortality (OR = 2.744, 95% CI 1.415–5.321, *P* = 0.003) and early hematoma expansion (OR = 2.607, 95% CI 1.711–3.974, *P* < 0.001) in patients with spontaneous intracerebral hemorrhage (Table 3). To explore whether the performance of different NCCT imaging markers in predicting poor prognosis of patients with intracerebral hemorrhage is superior to the irregular shape, we comprehensively interpreted the NCCT imaging markers, including the Island sign and Blend sign, in 627 spontaneous ICH patients included in our study. The predictive performance of different NCCT imaging markers for poor outcomes in ICH patients was analyzed with specificity and sensitivity, and the ROC curve was drawn. Our results demonstrated that irregular shape was better than other NCCT imaging markers for predicting the occurrence of unfavorable outcomes in ICH patients (area under the curve-AUC = 0.819, sensitivity 74.0%, specificity 78.7%, CI 0.781–0.856, Fig. 3A–C). Furthermore, in our study, the appearance of irregular shapes of the hematoma was a better predictor of poor patient functional outcome than the initial hematoma volume. (AUC = 0.808, Fig. 3D).

**Table 2:** Univariate associations with poor functional outcomes (mRS 3–6) and mortality and hematoma expansion.

Variable	90-day poor outcome			90-day mortality			HE		
	OR	95% CI	*P* value	OR	95% CI	*P* value	OR	95% CI	*P* value
Age (years)s	1.037	1.020–1.054	< 0.001	1.032	1.009–1.055	0.006	0.984	0.957–1.012	0.261
Sex (male)	0.988	0.678–1.438	0.948	0.975	0.568–1.673	0.926	0.352	0.153–0.811	0.014
History of hypertension	1.608	0.843–3.067	0.150	1.675	0.582–4.823	0.339	1.322	0.390–4.489	0.654
History of diabetes	1.229	0.698–2.161	0.475	1.196	0.560–2.553	0.643	0.940	0.353–2.502	0.901
First systolic BP (mmHg)	1.016	1.009–1.022	< 0.001	1.025	1.015–1.034	< 0.001	1.009	0.998–1.021	0.100
Heart rate	1.012	1.002–1.023	0.021	1.024	1.011–1.038	< 0.001	0.991	0.971–1.011	0.373
Temperature	1.769	0.915–3.421	0.090	2.120	0.906–4.962	0.083	0.657	0.179–2.412	0.527
Initial hematoma volume (mL)	1.043	1.033–1.054	< 0.001	1.039	1.029–1.048	< 0.001	1.025	1.012–1.038	< 0.001
IVH	3.283	2.258–4.772	< 0.001	3.576	2.125–6.018	< 0.001	1.071	0.547–2.097	0.842
Location of ICH	1.355	0.757–2.425	0.307	0.577	0.283–1.178	0.131	0.800	0.297–2.154	0.659
Midline shift	1.258	1.189–1.332	< 0.001	1.260	1.186–1.339	< 0.001	1.140	1.046–1.243	0.003
Admission INR	1.365	0.567–3.290	0.488	1.646	0.640–4.234	0.301	1.109	0.271–4.531	0.886
Admission PT (s)	1.036	0.950–1.131	0.423	1.059	0.966–1.160	0.221	0.999	0.858–1.162	0.985
Admission APTT, s	0.912	0.863–0.965	0.001	0.972	0.901–1.048	0.461	1.016	0.927–1.114	0.731
Admission D-Dimer, > 0.55 mg/L FEU	2.809	1.928–4.092	< 0.001	3.518	2.096–5.905	< 0.001	1.032	0.533–1.998	0.926
Admission fibrinogen (g/L)	1.062	0.949–1.188	0.294	0.969	0.721–1.154	0.721	0.626	0.413–0.949	0.027
Irregular sign	4.380	3.003–6.389	< 0.001	5.233	2.991–9.155	< 0.001	3.776	1.950–7.312	< 0.001
GCS score on admission	0.900	0.842–0.962	0.002	0.862	0.790–0.940	0.001	0.958	0.851–1.078	0.474
Surgery	3.054	2.060–4.527	< 0.001	0.668	0396–1.129	0.132	7.674	3.941–14.942	< 0.001

APTT = activated partial thromboplastin time, BP = blood pressure; CI = confidence interval; GCS = Glasgow Coma Scale; ICH = intracerebral hemorrhage; INR = international normalized ratio; IVH = intraventricular hemorrhage on presentation; mRS = modified Rankin Scale; NIHSS = NIH Stroke Scale; PT = prothrombin time. Values are n (%), mean ± SD, or median (interquartile range).

**Table 3:** Multiple associations with Poor Functional Outcome (mRS 3–6) and Mortality and Hematoma expansion.

Variable	90-day poor outcome			90-day mortality			HE		
	Adjusted OR	95% CI	*P* value	Adjusted OR	95% CI	*P* value	Adjusted OR	95% CI	*P* value
Age (years)	1.052	1.031–1.074	< 0.001	1.034	1.004–1.065	0.026	0.978	0.947–1.011	0.188
First systolic BP (mmHg)	1.009	1.000–1.017	0.045	1.014	1.002–1.025	0.019	1.008	0.996–1.020	0.211
Heart rate	1.012	0.998–1.027	0.095	1.021	1.004–1.037	0.014	0.990	0.969–1.012	0.375
Irregular sign	3.216	2.023–5.112	< 0.001	2.689	1.376–5.255	0.004	2.527	1.211–5.275	0.014
Midline shift	1.070	0.982–1.165	0.121	1.041	0.944–1.147	0.421	1.007	0.878–1.155	0.923
Initial hematoma volume (mL)	1.040	1.010–1.040	0.001	1.023	1.009–1.038	0.002	1.020	1.000–1.041	0.048
D-dimer, > 0.55 mg/L FEU	1.367	0.830–2.252	0.219	1.548	0.767–3.124	0.223	1.209	0.540–2.706	0.644
Admission APTT (s)	0.975	0.911–1.044	0.467	1.098	1.007–1.199	0.035	1.048	0.946–1.160	0.370
Admission fibrinogen (g/L)	1.056	0.939–1.188	0.363	0.791	0.563–1.110	0.175	0.612	0.391–0.957	0.031
GCS score on admission	0.935	0.862–1.014	0.103	0.869	0.781–0.967	0.010	0.989	0.871–1.122	0.860
IVH	2.002	1.257–3.189	0.003	1.874	0.961–3.653	0.065	0.806	0.372–1.744	0.583

BP = blood pressure; CI = confidence interval; GCS = Glasgow Coma Scale; ICH = intracerebral hemorrhage; IVH = intraventricular hemorrhage on presentation; mRS = modified Rankin Scale. Values are n (%), mean ± SD, or median (interquartile range).

**Figure 4:**
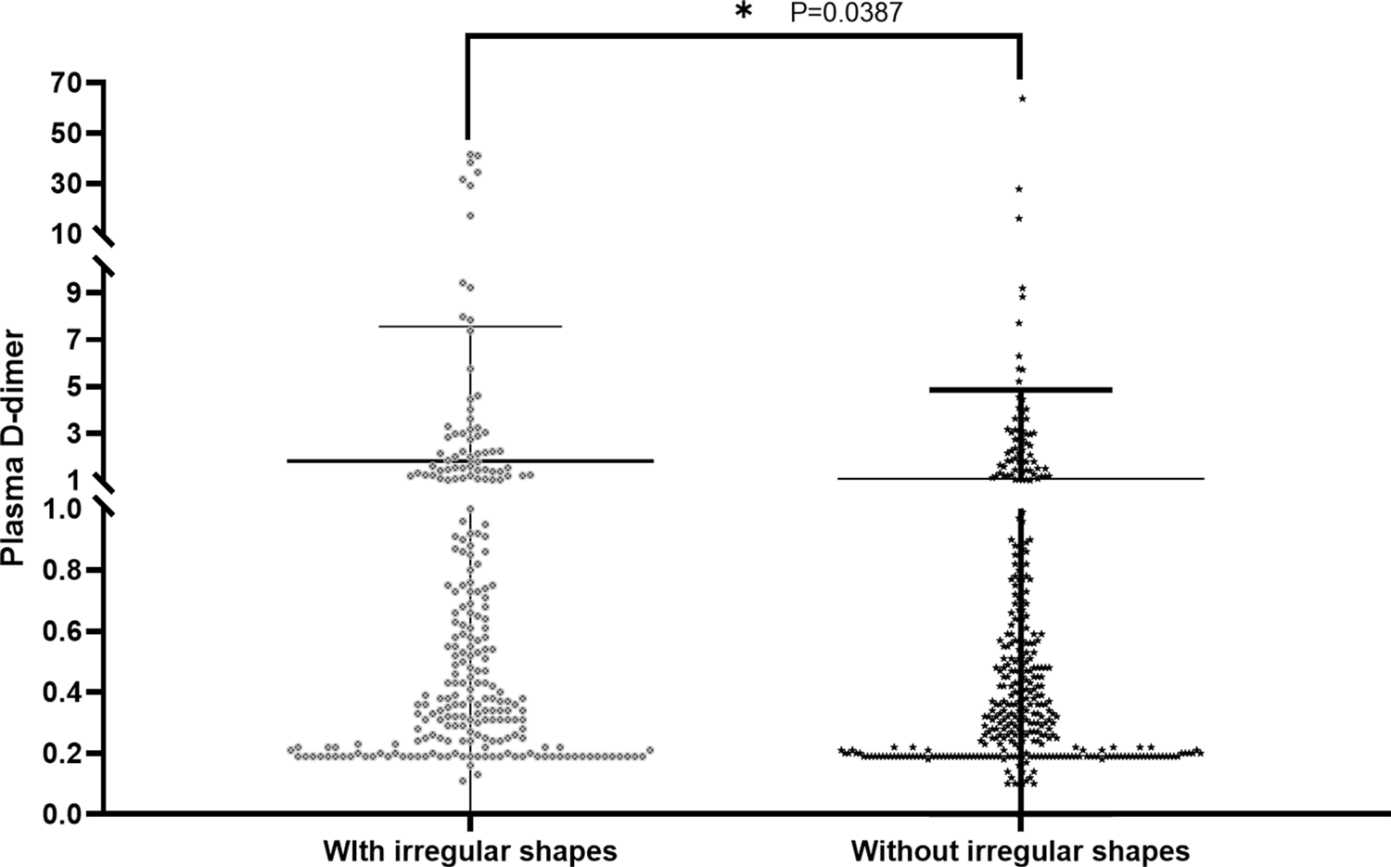
Kaplan–Meier curve analysis showing that the overall survival of patients with spontaneous intracerebral hemorrhage with irregular shapes in intracranial hematoma as worse than that with stable hematoma.

**Figure 5:**
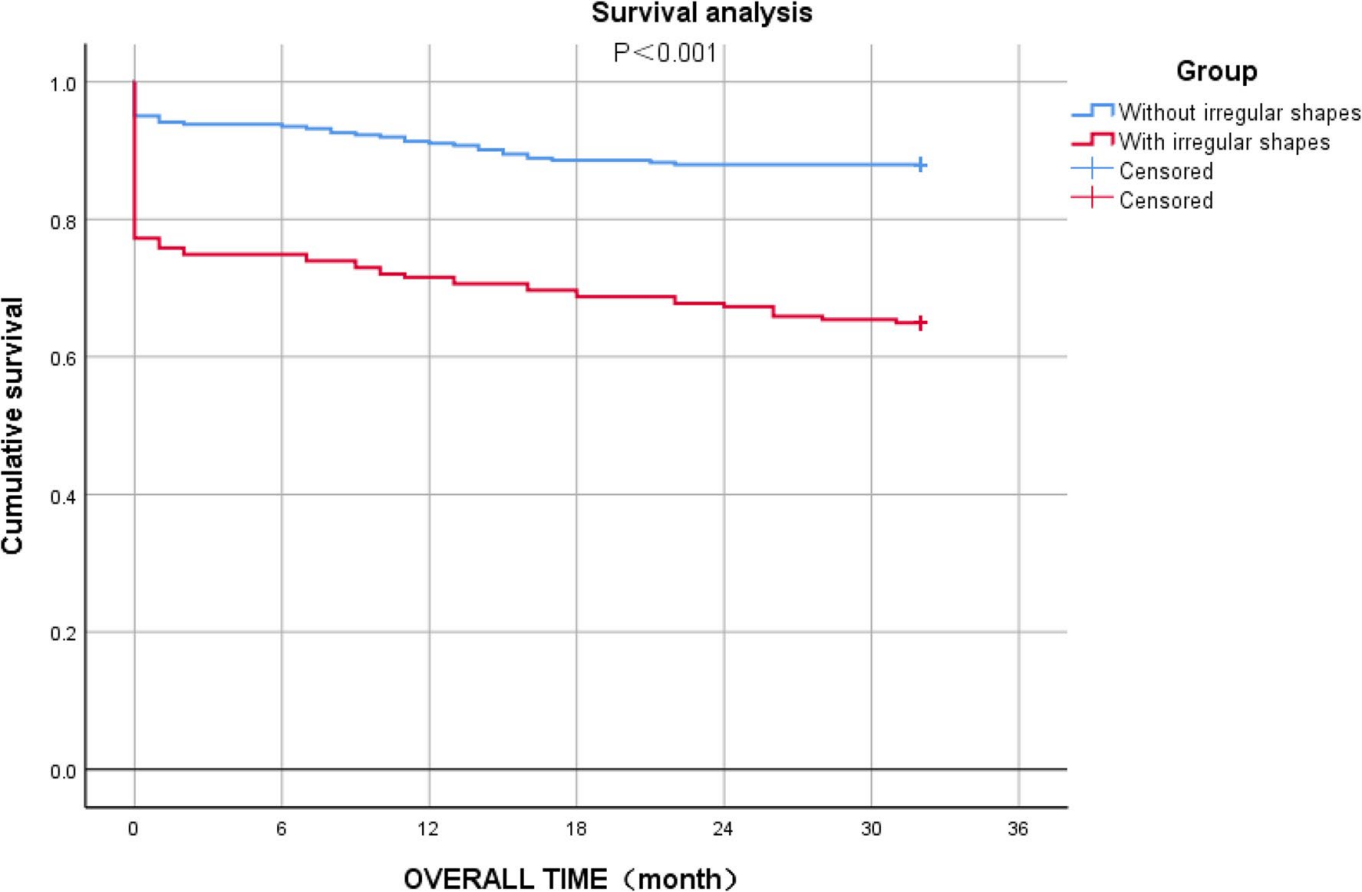
Scatter plot showing the relationship between the irregular shape and plasma D-dimer expression level. The appearance of irregular shapes in patients is often accompanied by an increase in plasma D-dimer expression on admission.

All patients will be followed up until death or the end of the study. A total of 535 patients were included in the Kaplan–Meier survival analysis, except for 92 patients whose death information was missing. The longest follow-up among all patients reached 32 months, with a median of 20 months, and by the end of the study on September 30, 2020 (Fig. 4). The results showed that the irregular shape of the hematoma was significantly associated with shorter survival of patients with cerebral hemorrhage (*P* < 0.001). In addition, in the data analysis, we also found that the appearance of irregular shapes was accompanied by altered plasma D-dimer expression levels, so we speculated whether the generation of irregular shapes resulted from the increased plasma D-dimer expression. In the scatter plot, plasma D-dimer expression levels were significantly correlated with the appearance of irregular shapes (*P* = 0.039, Fig. 5).

## Discussion

Our statistical results shows that irregular shape has predictive significance for the prognosis of patients with ICH. Though island sign, blend sign and initial hematoma volume also have some prognostic value for ICH, the predictive value of irregular shape for the prognosis of ICH is still more significant comparing with them.

As a severely disabling neurological disease, spontaneous intracerebral hemorrhage (ICH) is receiving more and more attention on how to quickly and accurately predict the occurrence of its adverse consequences, and then help clinicians to grade and manage patients. Regarding imaging, although there remain some controversies surrounding the clear definition and formation mechanism of the spot sign on CTA, it has been recognized as a well-established imaging marker with 51%–91% sensitivity and 58%–89% specificity for predicting the occurrence of hematoma expansion in the short term in ICH patients^7,15^, and the spot sign has also been identified as an independent predictor of poor prognosis in intracerebral hemorrhage. However, the need for contrast media for some patients (e.g., kidney failure, contrast allergy) limits CTA availability. The extravasation of contrast medium that occurs during CTA procedures may contribute to the occurrence of rebleeding^16^, posing an additional risk for some patients, which is not routinely performed in emergencies. Therefore, researchers have gradually explored the predictive value of NCCT markers, including the blend sign, irregular shape, and island sign, for intracerebral hemorrhage expansion, and the results showed equally good predictive performance^11,17^. Qi et al., in a retrospective analysis of 252 patients with intracerebral hemorrhage, showed that the island sign was an independent predictor of hematoma enlargement and poor outcomes in patients with ICH^18^. In a study conducted by Zhang et al. 1111 patients with ICH were recruited, and it was found that blend signs could help doctors grade the management of patients with intracerebral hemorrhage at admission^19^. In this study, we also analyzed the performance of different NCCT image markers in predicting poor functional prognosis in patients with cerebral hemorrhage, and the results showed good performance. In which the predictive power of irregular shapes was the best. In addition, many studies have demonstrated the significant value of hematoma volume for the prediction of prognostic situations in patients with intracerebral hemorrhage^20–23^. In our study, initial hematoma volume predicted poor functional outcomes in patients than irregularly shaped hematomas.

In a previous study, Moratti et al. established a scoring system to predict the instability of the hematoma based on three indicators: presence or absence of the mixed sign, time to the appearance of the mixed sign, and reduction in the density of the hematoma at presentation^24^. However, these studies did not directly use the irregular shape of the hematoma to evaluate the prognosis of patients and did not explore the cause of NCCT imaging hallmarks.

The overall survival (OS) of patients with irregular shapes in our study was significantly shorter than that of patients without irregular shapes. During a follow-up of up to 32 months for all patients included in this study, we recorded in detail the survival performance as well as the functional outcome status (mRS score) of patients with intracerebral hemorrhage after discharge. To our knowledge, this is the study with the longest follow-up time for a large number of patients among the relevant studies. Although we found death information for 20 of these patients by asking community hospitals or inquiring about patient death records, the survival status of 92 patients with ICH after discharge was unavailable.

The reason for the formation of irregular shape imaging signs was not deeply investigated in a large number of previous studies. In the present study, we showed for the first time that the appearance of irregular shapes was significantly associated with the elevation of plasma D-dimer (> 0.55 mg/L FEU). Plasma D-dimer has been shown to have good performance in the prediction of poor outcomes and mortality in patients with spontaneous ICH^12,25,26^. Acute brain injury resulting from hematoma formation is closely related to coagulation activation, while D-dimer, the final product of coagulation, is a fibrin degradation product released from plasmin degrading fibrin monomers^27^, and its expression quantity reflects the activation level of the systemic coagulation response. The massive release of thrombin, which induces inflammatory reactions in the perivascular tissues and then damages the blood–brain barrier (BBB), may lead to early hematoma expansion^28,29^. However, there was no significant statistical association between plasma D-dimer levels and hematoma expansion at admission in this study, which may be related to sample size and systematic error.

Our study has certain limitations. First, the patients included in this study were all hospitalized in the stroke emergency center of our hospital. Although other hospitals in the city have also established stroke centers, they are small and have low comprehensive diagnosis and treatment capacity, so patients with severe disease will more likely be treated in our hospital. This may contribute to a worse functional prognosis and even higher mortality in the patient population included in this study.

Second, some patients may have delayed going to the hospital for treatment, so the delay in time from symptom onset to admission and CT scans may have affected some variables in this study, for example, hematoma expansion, as they may have stabilized before the first CT examination. Previous studies have demonstrated that hematoma expansion is more likely to occur within the first hour of intracerebral hemorrhage symptom onset^30^. However, it is very difficult to obtain a precise judgment of the specific time of onset for each patient with intracerebral hemorrhage.

Finally, all patients in this study were Chinese, and no other races were included in the study, so the findings may not indicate the actual incidence in other regions. Although the morbidity of patients with nontraumatic ICH is directly due to cerebrovascular rupture, the main causes of ICH are not the same in Western countries as in Asia. Chronic hypertension is a major cause of ICH in China and other Asian countries, but cerebrovascular amyloidosis and oral anticoagulants play a more important role in the occurrence of ICH in Western countries^3,31–33^. This may be because aging is more severe in Western developed countries, resulting in the deposition of amyloid-β peptide causing degeneration of cerebral vessels and the need for more anticoagulant or antiplatelet drug therapy in the elderly to combat the occurrence of ischemic diseases^34^. Hypertension can lead to massive expression of vascular extracellular matrix such as laminin and fibronectin, which will destroy the structure of cerebrovascular, leading to vascular sclerosis as well as increased permeability^35,36^. This is also the reason why cerebral hemorrhage shows a strong association with hypertensive disorders^37^. In China, the incidence of hypertension is 2.5 times higher than that in the United States, and more than 80% of stroke patients have been diagnosed with hypertension^36,38^. In addition, Chinese patients were more inclined to use a single blood pressure control drug when treating hypertension, and the proportion of polypharmacy combination therapy was only 21. 9%, which would result in less effective prevention and control of hypertension^39,40^. Therefore, the difference in the incidence of hypertension and the difference in medication strategies may be important factors leading to the difference in the causes of ICH between China and Western countries. Future studies using larger samples are needed to support our findings, which will help clinicians find a more rapid and accurate method to judge the occurrence and progression of patients with ICH.

## Conclusion

CT irregular shape has an excellent performance in predicting functional outcomes in patients with spontaneous intracerebral hemorrhage. As an imaging marker of NCCT, the irregular shape can help doctors identify patients with rapid disease progression, poor prognosis, and high mortality. Moreover, this is the first study to confirm the statistical significance of D-dimer in predicting irregular shapes and has excellent potential to further explore the pathophysiological mechanism of D-dimer in hematoma enlargement. To improve prediction accuracy and sensitivity, plasma D-dimer level increases can be combined with other cerebral hemorrhage-related factors in subsequent studies.

## Supplementary Information

Supplementary Figure 1.

Supplementary Legends.

## Data Availability

The data that support the findings of this study are available from the corresponding author upon reasonable request.

## Abbreviations

CI: Confidence interval
CT: Computed tomography
CTA: Computed tomography angiography
GCSs: Glasgow Coma Scale score
HE: Hematoma expansion
ICH: Intracerebral hemorrhage
IQR: Interquartile range
mRS: Modified Rankin Scale
NCCT: Non-contrast computed tomography
OR: Odds ratio
OS: Overall survival
SD: Standard deviation
