# Supplementary figures and images for: Irregular shape as an independent predictor of prognosis in patients with primary intracerebral hemorrhage
Source: Sci Rep. 2022 May 20;12:8552. doi: 10.1038/s41598-022-12536-3 (PMC9123162; doi:10.1038/s41598-022-12536-3)

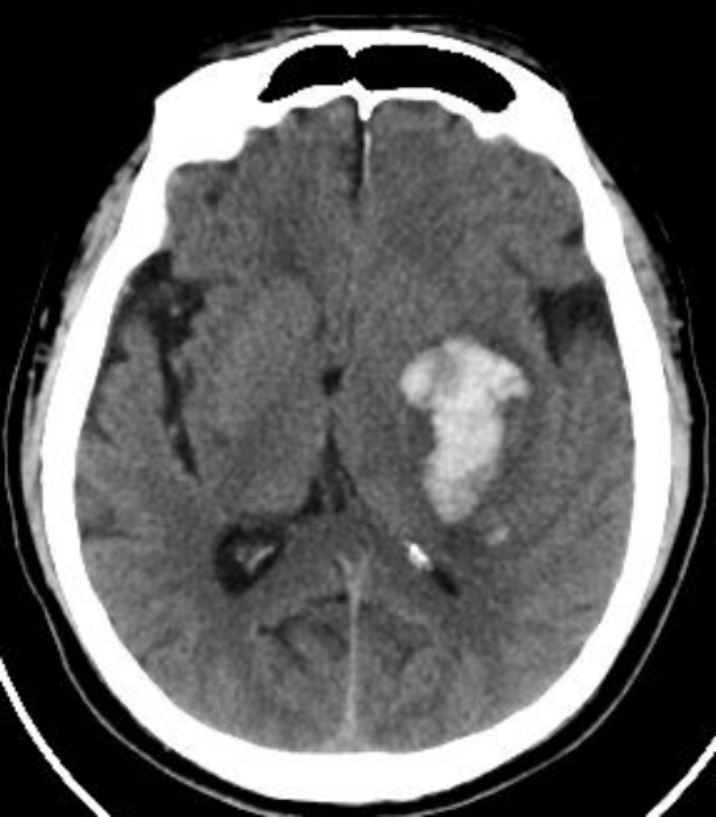

Supplement: Supplementary file 1 — Supplementary Figure 1. [file 41598_2022_12536_MOESM1_ESM.tiff]
