# Supplementary material for: Irregular shape as an independent predictor of prognosis in patients with primary intracerebral hemorrhage
Source: Sci Rep. 2022 May 20;12:8552. doi: 10.1038/s41598-022-12536-3 (PMC9123162; doi:10.1038/s41598-022-12536-3)
Supplement: Supplementary file 2 — Supplementary Legends. [file 41598_2022_12536_MOESM2_ESM.docx]

**Figure Legends**

**Supplemental Figure** Irregular shapes were defined as the presence of two or more connected or separated irregular hematoma at the edge of the hematoma on the axial section with the largest hematoma cross-sectional area.
